# Menstrual status and bone health in athletes from different sports – a retrospective analysis

**DOI:** 10.1186/s13102-026-01781-y

**Published:** 2026-06-09

**Authors:** Gina F. Øistuen, John O. Osborne, Jorunn Sundgot-Borgen, Dionne A. Noordhof, Madison Y. Taylor, Ida Heikura, Trent Stellingwerff, Sara A. Solheim, Helge E. Lundberg, Therese F. Mathisen, Mette Hansen, Mikkel Oxfeldt, Line B. Dalgaard, Erik P. Andersson, Klavs Madsen, Kristin L. Jonvik

**Affiliations:** 1 Department of Physical Performance, Norwegian School of Sport Sciences, Oslo, Norway; 2 School of Sport Sciences, Faculty of Health, The Arctic University of Norway, Tromsø, Norway; 3 Sunshine Coast University Hospital, Birtinya, Australia; 4 School of Health, University of the Sunshine Coast, Sippy Downs, Australia; 5 School of Exercise and Nutrition Sciences, Queensland University of Technology, Brisbane, Australia; 6 Department of Sports Medicine, Norwegian School of Sport Sciences, Oslo, Norway; 7 Centre for Elite Sports Research, Department of Neuromedicine and Movement Science, Norwegian University of Science and Technology, Trondheim, Norway; 8 Canadian Sport Institute - Pacific, Victoria, BC Canada; 9 Exercise Science, Physical and Health Education, University of Victoria, Victoria, BC Canada; 10 Norwegian Doping Control Laboratory, Oslo University Hospital, Oslo, Norway; 11 Faculty of Health, Welfare and Organisation, Østfold University College, Fredrikstad, Norway; 12 Department of Public Health, Section of Sport Science, Aarhus University, Aarhus, Denmark; 13 Department of Health Sciences, Swedish Winter Sports Research Centre, Mid Sweden University, Östersund, Sweden

**Keywords:** Relative Energy Deficiency in Sports, Menstrual disturbances, Menstrual cycle, Hormonal contraceptives, Bone Mass, Impact Sport

## Abstract

**Background:**

Relative energy deficiency in sport (REDs) is linked to low energy availability, menstrual disturbances (MD) and low bone mineral density (BMD; Z-score < -1.0). The prevalence of these symptoms across sports and their interrelationships requires further investigation. This study aimed to 1) investigate the prevalence of severe MD and low BMD among athletes from different sports, 2) compare BMD Z-scores at lumbar spine, total hip and whole-body between regularly menstruating (RM), severe MD and hormonal contraceptive (HC) users, and 3) examine factors related to BMD among endurance athletes, including menstrual status, athletic caliber and sport-specific loading characteristics.

**Methods:**

Retrospective analysis of cross-sectional data from seven projects included 411 women from 34 sports (endurance: n = 257; non-endurance: n = 154). BMD was assessed using dual-energy X-ray absorptiometry. Menstrual status was defined by the three-step method, cycle tracking or questionnaire, and categorized as RM, severe MD or HC. Endurance sports were further grouped by loading characteristics (very low, low and medium).

**Results:**

Among non-HC users (51%), the prevalence of severe MD was 37% in endurance, 34% in ball sport and 36% in power- and technical- sports. BMD was not different between menstrual status groups at either lumbar spine (*P* = 0.334), total hip (*P* = 0.079) or whole-body (*P* = 0.164). Endurance athletes had lower lumbar spine BMD Z-scores than non-endurance athletes (-0.0 ± 1.0 vs 1.2 ± 1.2, *P* < 0.0001), with a higher prevalence of low BMD (17% vs 2%, *P* = 0.015). Total hip BMD Z-scores were lower in endurance than non-endurance athletes (0.6 ± 0.9 vs 1.4 ± 1.2, *P* < 0.05 for age < 27). Among endurance athletes, neither loading characteristics nor athletic caliber were associated with BMD. However, endurance athletes with severe MD had lower total hip BMD Z-scores compared to RM athletes (*P* = 0.008) and HC users (*P* = 0.021).

**Conclusions:**

The prevalence of severe MD did not differ between sport types. Overall, BMD Z-scores did not differ across menstrual status categories. However, endurance athletes had lower BMD, with total hip BMD being lower in those with severe MD. Heterogenous menstrual assessment methods across the projects included in this study may have impacted the results. Future research should integrate comprehensive REDs assessments including detailed data on menstrual status, training history, bone-loading impact and injuries in female athletes from different sports.

**Supplementary Information:**

The online version contains supplementary material available at 10.1186/s13102-026-01781-y.

## Introduction

Menstrual disturbances (MD) and low bone mineral density (BMD) are symptoms of relative energy deficiency in sport (REDs). REDs is a highly prevalent condition among female athletes (23–80%), depending on the type of sport and athletic caliber [27] and is characterized by impaired physiological and/or psychological functioning caused by problematic (prolonged or severe) low energy availability (LEA). LEA is defined as insufficient energy intake to cover both the energy expenditure of exercise and the energy required for essential physiological functions [27].

MD can be distinguished as subtle or severe. Subtle MD includes absence of ovulation (anovulation), low mid-luteal phase progesterone value or short luteal phase length (luteal phase defect). Severe MD can be defined as marked disturbances of menstrual function, including a menstrual cycle length > 35 days (oligomenorrhea), absence of menarche by age 15 (primary amenorrhea) and the absence of menses for three or more months in non-pregnant women with past menses (secondary amenorrhea). A recent meta-analysis based on 60 studies in 6380 female athletes [41], found a mean prevalence of 7% primary amenorrhea, 16% secondary amenorrhea and 25% oligomenorrhea. The prevalence of severe MD is particularly high in leanness sports, where body mass is considered crucial for performance (Gimunova et al., [13, 44]. Regularly menstruating (RM) women, on the other hand, have a menstrual cycle length of 21–35 days, and are defined as eumenorrheic if they have confirmed ovulation and progesterone concentrations above 16 nmol/L in mid-luteal phase (Elliot-Sale et al., 7. The presence of MD can only be determined in women not using hormonal contraceptives (HC) because HC use can mask a possible MD. In the Nordic countries, HC are used by 30–49% of women from the general population [12, 23, 31]. In contrast, studies investigating HC use in athletic populations have reported that 29–68% of female athletes use HC, with large differences reported between sports and countries [6, 8, 21].

Estrogen and progesterone have beneficial effects on bone resorption and formation, respectively, and are important for maintaining good bone health in women [38]. The combination of LEA and estrogen deficiency has been shown to reduce bone turnover and bone formation compared with women who are estrogen deficient but energy replete [40]. Furthermore, dynamic weight-bearing activity is suggested as the most effective form of exercise for bone health [42], especially when it generates high mechanical strain with both great magnitude and rapid loading rates, i.e., high impact (Weeks et al., 46. Both mechanical load and menstrual status are suggested as two crucial factors for bone formation, as lower BMD is reported in athletes from lower impact sports with severe MD compared to both athletes from high impact sports and regularly menstruating non-athletes [1, 29]. In addition, endurance athletes have been reported to have lower BMD compared to athletes participating in ball sports and power sports [17],Scofield et al., 37, [42]. This may be explained by the higher mechanical loading in power sports, but it could also be related to the high energy demand in endurance sports, increasing the risk of LEA and the large benefit of having a lower body mass in weight bearing endurance sports.

Although the detrimental effects of MD on BMD are well-documented [1, 29], there is limited knowledge regarding the prevalence of these symptoms in the same individuals, their interrelationship and the influence of type of sport and athletic caliber. As such, the purpose of the current study was to: (1) investigate the prevalence of severe MD and low BMD in athletes from different sports (2) compare BMD Z-scores at lumbar spine, total hip and whole-body level between women with RM, severe MD and those using HC,and (3) investigate which factors influence BMD among endurance athletes, such as menstrual status, athletic caliber, and sport-specific loading characteristics.

## Methods

### Study design

This retrospective analysis included cross-sectional data on menstrual status and BMD from seven different projects, and all data were collected from baseline measurements prior to any intervention. Data were collected between 2018–2025 in Norway (project 1–5), Denmark (project 6) and Canada (project 7). See Table 1. In brief, to determine menstrual status, the three-step method (project 1, 3 and 5), athlete tracking of MC length during 12 months (project 2) or 3 months (project 6), and Low Energy Availability in Females Questionnaire (LEAF-Q, project 4 and 7) was used. Paired BMD data was provided in project 1–5 and 7 but were not available in project 6.

**Table 1:** Overview of the cross-sectional projects included

Project number	Project name	Country	Project aim	Highly relevant inclusion criteria	N	Type of data (method)	Reference
1	Menstrual Cycle Phase has no Influence on Performance-Determining Variables in Endurance-Trained Athletes: The FENDURA Project	Norway	To investigate the effect of the MC and endogenous sex hormone concentrations on performance-determining variables in three distinct MC phases in endurance-trained females	Endurance trained female athletes with MC length between 21–35 days for the last six months. Age: 17–40 yr. Recruited for non sMD	31	Z-score whole body, lumbar spine and total hip (DXA; GE Lunar Prodigy) Menstrual status (Three step method for MC verification^b^)	PMID: 38600646
2	Propionebacterium freudenreichii—a diet component with promising effect on bone health in athletes; A randomised clinical trial	Norway	To investigate the role of nutrition in relation to REDs-related markers, bone health, muscle strength, and aerobic capacity in young elite male and female cross-country and biathlon skiers	Competitive cross-country skiers and biathletes. Age: 17–30 yr	26	Z-score whole body, lumbar spine and total hip (DXA; GE Lunar Prodigy) Menstrual status (Athlete tracking of average MC-length the last year)	Under review
3	Test–retest reliability of strength, power, agility, and sprint performance in female team handball players	Norway	To assess the test–retest reliability of athletic performance tests in a cohort of trained Norwegian female handball players, as well as a sub-analysis of the test–retest reliability for naturally menstruating players	Competitive female team handball athletes either using HC or with MC length between 21–35 days for the preceding two months. Age: 16–29 yr. Recruited for non sMD	16	Z-score whole body, lumbar spine and hip (DXA; GE Lunar Prodigy) Menstrual status (Three step method for MC verification^b^)	PMID: 39645439
4	Mental health and symptoms of REDs in young biathlon athletes	Norway	To examine the prevalence of mental health symptoms, LEA, and REDs among biathletes, to explore age- and sex-related patterns, and to investigate whether REDs-related health symptoms and training characteristics in early season are associated with performance at the end-of-season national championship	Competitive biathletes of age range. Age: 18–20 yr	11	Z-score whole body, lumbar spine and total hip (DXA; GE Lunar Prodigy) Menstrual status (LEAF-Q^c^)	ClinicalTrials.gov ID: NCT06487260, registration date: 5th July 2024
5	Resistance Training as Confounder on Steroid and Endocrine Profiles in Serum	Norway	To evaluate non-analytical factors that may influence the markers in the serum steroid profiles and endocrine profiles, which are indirect methods to detect doping with anabolic androgenic steroids and growth hormone, respectively	Females practicing weightlifting with a MC-length between 21–35 days for 3 or more MCs. Age: 18–40 yr. Recruited for non sMD	2	Z-score whole body, lumbar spine and total hip (DXA; GE Lunar Prodigy) Menstrual status (Three step method for MC verification^b^)	https://www.wada-ama.org/en/resources/scientific-research/low-energy-availability-and-resistance-training-confounders-steroid
6	Hormonal Contraceptive Use, Menstrual Dysfunctions, and Self-Reported Side Effects in Elite Athletes in Denmark	Denmark	To identify the prevalence of hormonal HC use, menstrual cycle disturbances, and self-perceived physical and emotional symptoms related to the menstrual cycle/pill cycle in elite female athletes	Female elite athletes associated with the national organization for the support of elite sport in Denmark Age: 16–51 yr.^a^	178	Menstrual status (Athlete tracking of average MC-length the last 3 months) No DXA data available	PMID: 32957078
7	Application of the IOC Relative Energy Deficiency in Sport (REDs) Clinical Assessment Tool version 2 (CAT2) across 200 + Elite athletes	Canada	To develop and evaluate the utility and validity of a new clinical assessment tool (CAT2) for the screening and diagnosis of relative energy deficiency in sport (REDs) in Canadian elite female and male athletes	Currently training and competing in Olympics/Paralympics sports and residing in Canada. Age: > 15 yr.^a^	158	Z-score whole body, lumbar spine and total hip (DXA; Hologic Horizon, GE Lunar Prodigy & GE Lunar iDXA) Menstrual status (LEAF-Q^c^)	PMID: 39164063

*Abbreviations*: *N* number of participants, *sMD* severe menstrual disturbances, *HC* hormonal contraceptives, *MC* menstrual cycle, *DXA* Dual-energy X-ray absorptiometry, *LEAF-Q* Low Energy Availability in Females-Questionnaire, *PMID* PubMed identification number

^a^Participants ≥ 40 yr were excluded

^b^Three step method for menstrual cycle (MC) verification [35]

^c^Low Energy Availability in Females Questionnaire [25]

### Participants

**Fig. 1:**
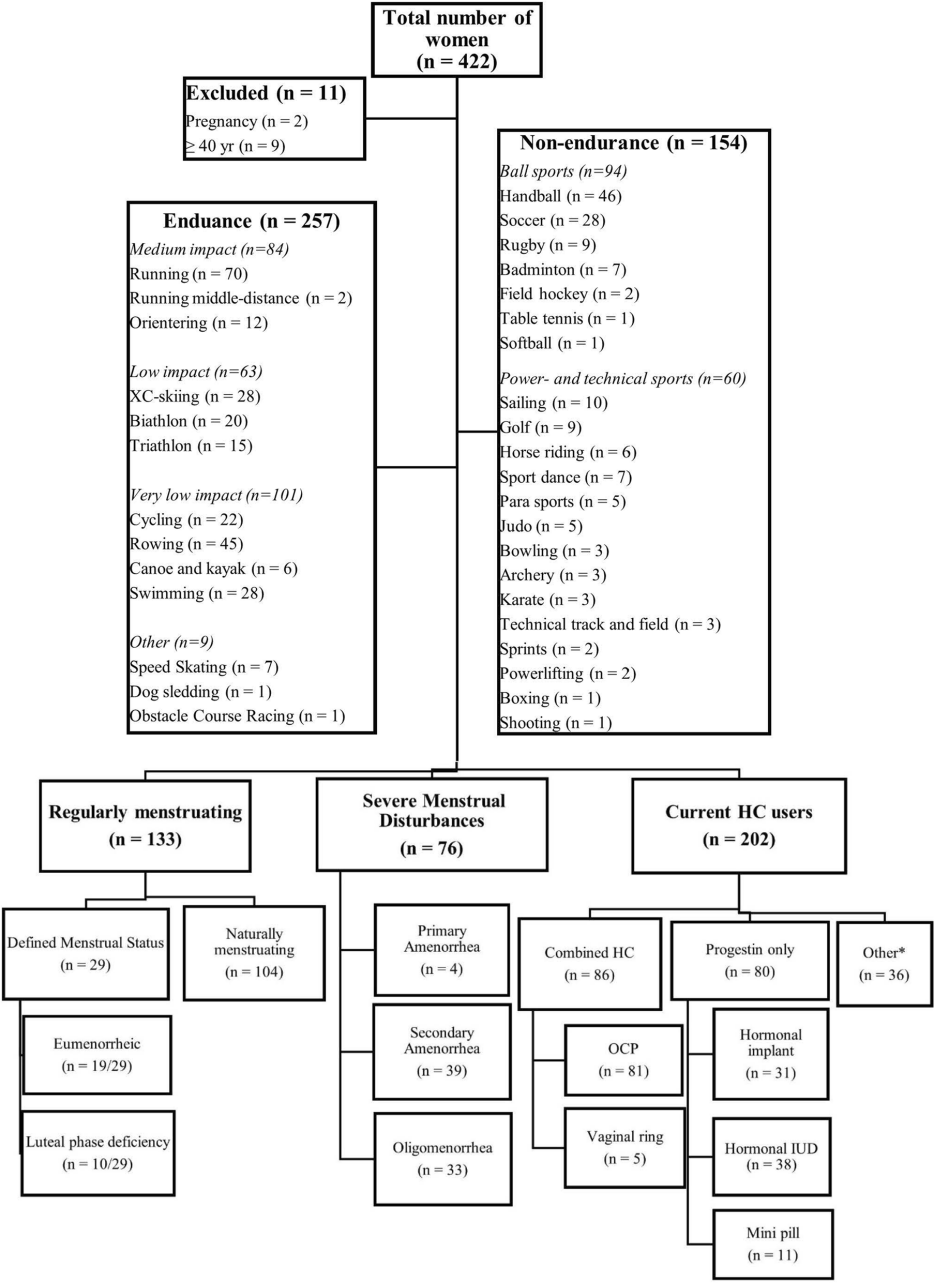
Overview of participants included grouped into sport categories and menstrual status. HC, Hormonal contraception; OCP, Oral contraception pill; IUD, Intrauterine device. ^*^ Type of HC pill not specified

The dataset included 422 women. Eleven participants were excluded due to pregnancy or age ≥ 40 years to minimize confounding from perimenopausal changes. In total, 411 healthy women between 16 and 40 years were included in the final analyses, of which all had data on menstrual status, and 221 had paired BMD data. The participants’ athletic caliber was classified as Tier 1 (*n* = 2), Tier 2–3 (*n* = 236) or Tier 4–5 (*n* = 173) according to the Participant Classification Framework proposed by McKay et al. [24]. In total, the participants represented 34 different sports which were grouped as either endurance (*n* = 257) or non-endurance sports (*n* = 154), whereof ball sports (*n* = 96) and power- and technical- sports (*n* = 60) (Fig. 1). Endurance sports were defined based on prolonged duration of the sport event (> 30 min) with goal of improving aerobic capacity [45].

### Definition of menstrual status

Menstrual status was defined by different methods within each of the seven cross-sectional projects, all using the menstrual status categorization explained below and in Table 1. In five of seven projects, menstrual status was defined based upon multiple cycles. Project 1 (*n* = 31), 3 (*n* = 16) and 5 (*n* = 2) provided data from 1–3 months of prospective menstrual tracking using the three-step method [35], including calendar-based counting, urinary ovulation testing and serum progesterone sampling during the mid-luteal phase. Project 2 (*n* = 26) and 6 (*n* = 178) provided the average MC length recorded during 12 or 3 months of athlete tracking, respectively. Project 4 (n = 11) and 7 (n = 158) provided participants responses from the LEAF-Q questionnaire, i.e. average cycle length, history of menstrual disturbances [25]. *Menstrual status* was categorized into three main groups: (1) RM was defined as a regularly menstruating with a menstrual cycle length between 21–35 days. Subtle MD could be included here due to lack of ovulation detection and any assessment of luteal phase defects. (2) Severe MD included secondary amenorrhea (absence of menses ≥ 3 months), primary amenorrhea (failure to reach menarche by age 15 years) and oligomenorrhea (menstrual cycle length > 35 days). Oligomenorrhea was included in the severe MD category according to REDs-related clinical classifications reflecting hypothalamic suppression [3]. (3) HC users included participants currently using either progestin-only or combined contraception (Fig. 1). Three of the included studies excluded women with MD or disturbed cycles from their data analysis, but they still have screened for MD and therefore those data have been included in the current manuscript.

### Bone mineral density

BMD was measured using dual-energy X-ray absorptiometry (DXA) in Norway (Project 1–5, Table 1, GE Lunar Prodigy, GE Healthcare, USA) and Canada (Project 7, Table 1, Victoria BC (Hologic Horizon W, USA); Vancouver BC (GE Lunar Prodigy, GE Healthcare, USA);Ontario and Quebec (GE Lunar iDXA, GE Healthcare, USA). Data on BMD Z-scores for whole body (*n* = 94), lumbar spine (*n* = 221) and/or total hip (*n* = 220) were reported. Some studies only collected data at one or two of the three skeletal sites, and thus the number of participants per site differs (Table 1). BMD Z-scores below −1.0 were classified as low BMD, in accordance with athlete-specific recommendations from the American College of Sports Medicine [28].

### Definition of sport impact

To achieve the third aim of this study, endurance athletes (*n* = 248) were categorized into three groups based on *loading impact characteristics*: very low, low and medium [46] (Fig. 1). Medium impact (*n* = 84) was classified as mainly weight-bearing activities: running, middle-distance running, and orienteering. Low impact (*n* = 63) was classified as a mix of activities with varying weight-bearing characters: XC-skiing, biathlon and triathlon. Very low impact activities (*n* = 101) included non-weight bearing activities: cycling, rowing, canoeing and kayaking, and swimming. Remaining endurance athletes (*n* = 9) were not categorized due to the unspecific loading impact of their sport, including speed skating, dog sledding and obstacle course racing.

### Data analysis

Chi-square tests were conducted to assess Aim 1 using IBM SPSS Statistics, Version 29 (IBM Corp., Armonk, NY). This included differences in the prevalence of RM, MD and HC, across the three sport categories (endurance, ball sport and power- and technical- sports), and differences in prevalence of low BMD per site between endurance and non-endurance athletes (combining ball sport and power- and technical- sports due to fewer data points for BMD from these sports).

Analyses for Aim 2 and Aim 3 were performed with R in the RStudio environment [34], using the following packages: *lme4* and *lmerTest* (mixed models), *emmeans* (post hoc comparisons), *performance* (diagnostics), *dplyr* (data wrangling), and *ggplot2* (visualisation).

Linear mixed-effects models were used to analyze BMD Z-scores at the whole body, total hip, and lumbar spine (L1–L4). For Aim 2, the primary fixed effect was *menstrual status* (levels: RM, severe MD, or HC use). For the first analysis of Aim 3, the primary fixed effect was *group* (levels: endurance and non-endurance]), while for the second analysis of Aim 3 (i.e., within endurance athletes only), fixed effects included *menstrual status*, *loading impact characteristics* (levels: very low; low; medium), and *athletic caliber* (levels: medium = Tier 2–3; high = Tier 4–5).

All models included *age* and *BMI* (both continuous) as covariates and *DXA machine* as a random intercept to account for potential measurement variability across different scanners. Potential interactions between the primary fixed effect and covariates were tested separately for inclusion using likelihood ratio tests, but none were retained in the final models. Post-hoc pairwise comparisons were corrected using the multivariate-*t* adjustment. For the endurance athlete models of Aim 3, comparisons and effect sizes were estimated at reference levels of *loading impact characteristics* (low) and *athletic caliber* (medium) to aid interpretability.

Model assumptions were checked via visual inspection of residuals and Q–Q plots, with homoscedasticity, linearity, and normality deemed acceptable. Variance inflation factors (VIFs) indicated no problematic multicollinearity. Statistical significance was set at α = 0.05. Data are presented as mean ± standard deviation or model-adjusted group means with 95% confidence intervals (95% CI) and Cohen’s *d* effect sizes, where relevant.

## Results

### Participants

Participant characteristics are presented in Table 2, grouped by type of sport.

**Table 2:** Participant characteristics into endurance, ball sport and power- and technical- sports

	**Endurance sports**	**Ball sports**	**Power- and technical- sports**	**Total**^**#**^
Age, Y (n)	22.8 ± 4.8 (*n* = 257)	21.8 ± 4.4 (*n* = 93)	24.0 ± 5.2^a^ (*n* = 59)	24.3 ± 4.9 (*n* = 409)
Height, cm (n)	171.9 ± 7.1^b^ (*n* = 236)	172.4 ± 7.2 (*n* = 94)	169.4 ± 4.9^a^ (*n* = 59)	172.6 ± 6.7 (*n* = 389)
Body mass, kg (n)	64.8 ± 9.3^a^ (*n* = 235)	67.8 ± 7.2 (*n* = 94)	63.0 ± 9.5^a^ (*n* = 59)	66.3 ± 8.8 (*n* = 388)
BMI, kg/m^2^ (n)	21.9 ± 2.2 (*n* = 235)	22.5 ± 2.9 (*n* = 94)	21.9 ± 2.6 (*n* = 59)	22.2 ± 2.0 (*n* = 388)
Athletic caliber, % (Tier 2–3/Tier 4–5)	59/41	63/37	41/56^*^	58/42

Mean ± SD for age, height, body mass and BMI. Significant difference (*P* < 0.05) from (a) ball sports and (b) power- and technical- sports

^*^There were also two athletes (3%) from power- and technical- sports that held Tier 1 level

^#^N varies with missing data per variable

### Menstrual status and sport

**Fig. 2:**
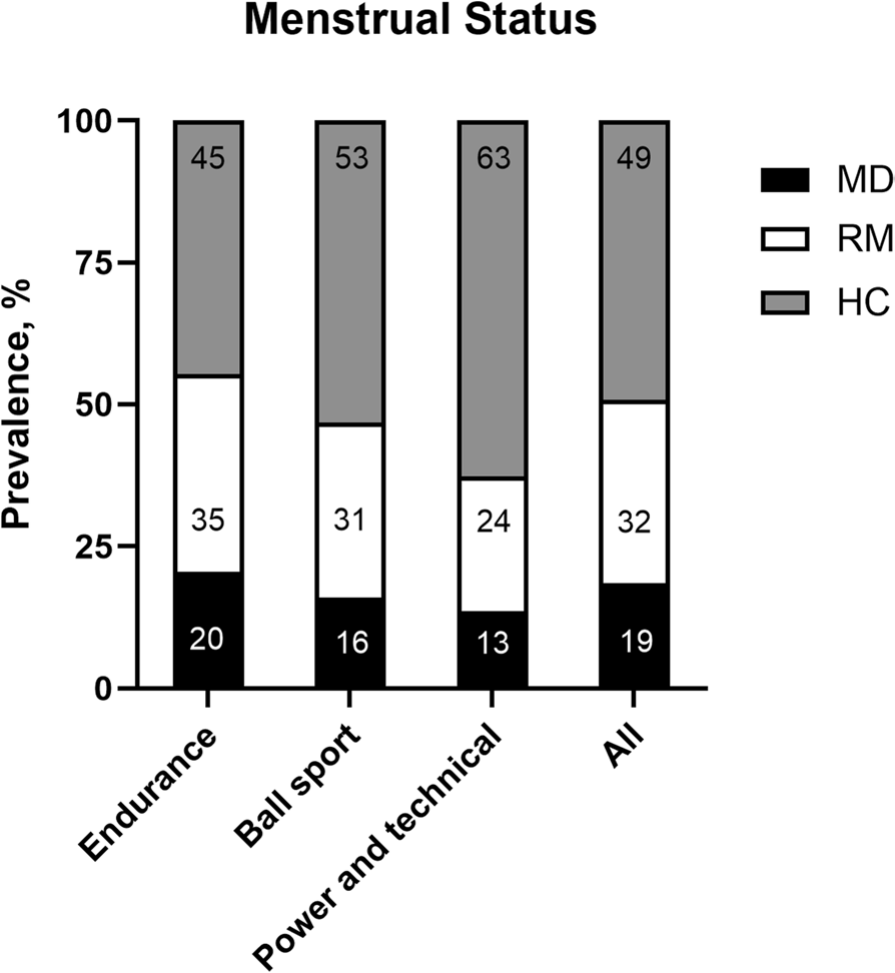
Prevalence of severe menstrual disturbances (MD), regularly menstruation (RM), and hormonal contraceptive use (HC) among endurance, ball sport, power- and technical- sports and all athletes combined

Figure 2 presents the prevalence of HC, RM and severe MD in different sport categories. In total, 49% of the participants used HC, with higher prevalence among power- and technical- sports (63%) than endurance (45%, *P* = 0.012), but not ball sports (53%, *P* = 0.25). HC use was significantly higher among Tier 4–5 (58%) compared to Tier 2–3 (43%, *P* = 0.002). The overall prevalence of severe MD is presented in Fig. 2, but a more informative perspective comes from examining the prevalence of severe MD among non-HC users (51%). Among non-HC users, the prevalence of severe MD was similar for endurance (37%), ball sports (34%), and power- and technical- (36%) sports (*P* = 0.94). The prevalence across sport categories did not change noteworthy when excluding the participants from the three studies that excluded women with severe MD (n = 30).

### Bone mineral density

**Fig. 3:**
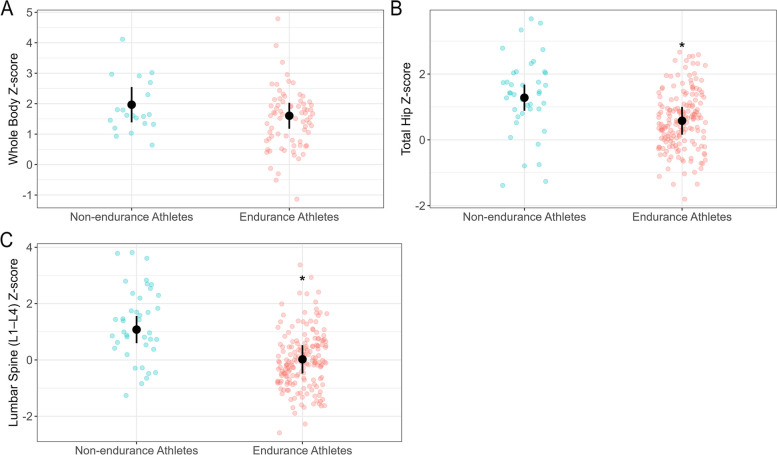
Bone mineral density by sport, for (**A**) whole-body, (**B**) total hip, and (**C**) lumbar spine. Individual colored points represent raw unadjusted participant data for endurance athletes and non-endurance athletes for (**A**) whole-body (*n* = 94), (**B**) total hip (*n* = 220), and (**C**) lumbar spine (*n* = 221). Black points indicate model-adjusted means for each group, with black vertical lines indicating 95% confidence intervals, derived from a linear mixed-effects model adjusted for age, BMI, and DXA machine (random intercept). Z-scores are unitless standardized values that represent the number of standard deviations above or below the population mean for age and sex. ^*^Indicate statistically significant differences (*P* < 0.05) between groups

Endurance athletes had a higher prevalence of low BMD at the lumbar spine compared to non-endurance athletes (17% vs 2%, *P* = 0.015). At the hip, there was no difference in prevalence of low BMD between endurance and non-endurance athletes (3% vs 5%, *P* = 0.64). Only one athlete had low BMD at the whole-body level. No main effect of group (i.e., endurance athletes vs non-endurance athletes) was found for whole-body BMD (*P* = 0.220, Fig. 3). However, there was a difference in lumbar spine BMD, with endurance athletes showing lower Z-scores (adjusted mean = 0.02, 95% CI: −0.48 to 0.53; n = 178) compared to non-endurance athletes (adjusted mean = 1.08, 95% CI: 0.59 to 1.56; *P* < 0.001; *d* = 1.04, 95% CI: 0.43 to 1.66; n = 40).

A significant interaction between *group* (endurance vs non-endurance athletes) and *age* was observed in the total hip BMD model (*P* = 0.013). In non-endurance athletes, total hip BMD declined significantly with age (slope = −0.081, 95% CI: −0.142 to −0.020; n = 40), whereas endurance athletes showed no significant age-related trend (slope = −0.002, 95% CI: −0.041 to 0.036; *n* = 177). Pairwise comparisons indicated that non-endurance athletes had significantly higher total hip BMD at younger ages (e.g., at age 20: adjusted mean difference = 0.95, 95% CI: 0.51, 1.39, *P* < 0.001*; d* = 1.04, 95% CI: 0.36, 1.71), but this group difference was no longer present from age 27 onwards (*P* = 0.063).

Lumbar spine BMD Z-scores were lower in those who reported to have a history of bone stress injury (n = 65, −0.04 ± 1.0) compared with those who reported no history of bone stress injury (n = 138, 0.37 ± 1.2, *P* = 0.018). No differences were seen for total hip (*P* = 0.29) or whole-body (*P* = 0.98) BMD Z-scores.

### Menstrual status and bone mineral density

**Fig. 4:**
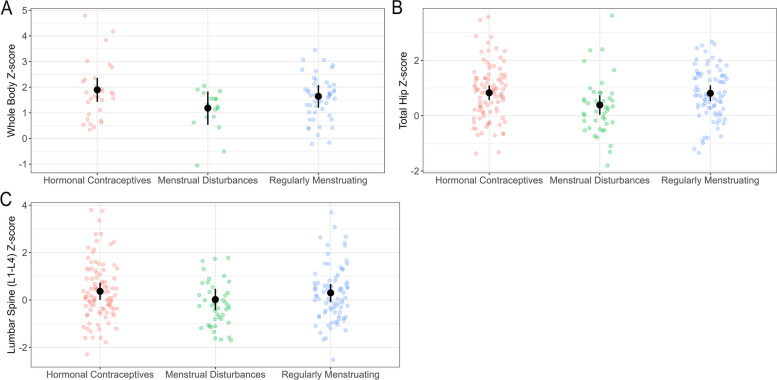
Bone mineral density by menstrual status, for (**A**) whole-body, (**B**) hip, and (**C**) lumbar spine. Individual colored points represent raw, unadjusted participant data for (**A**) whole-body (*n* = 94), (**B**) total hip (*n* = 220), and (**C**) lumbar spine (*n* = 221). Black points indicate model-adjusted means for each group with black vertical lines showing 95% confidence intervals, derived from a linear mixed-effects model adjusted for age, BMI, and DXA machine (random intercept). Z-scores are unitless standardized values representing the number of standard deviations above or below the population mean for age and sex. No statistically significant differences were observed between different menstrual status groups (*P* > 0.05)

BMD Z-scores did not differ significantly between menstrual status groups (RM vs severe MD vs HC users) at any anatomical site (Fig. 4). Specifically, no main effects for menstrual status group were observed for whole-body BMD (P = 0.164), total hip BMD (*P* = 0.079), or lumbar spine BMD (*P* = 0.334).

### Factors influencing BMD in endurance athletes

Among endurance athletes, menstrual status was significantly associated with total hip BMD (*P* = 0.007), but not whole-body BMD (*P* = 0.896) or lumbar spine BMD (*P* = 0.123) (Supplemental Fig. 1). Endurance athletes with severe MD had significantly lower total hip BMD Z-scores (adjusted mean = 0.18, 95% CI: −0.37 to 0.72; *n* = 33) compared to those with RM (adjusted mean = 0.81, 95% CI: 0.34 to 1.280; *P* = 0.008; *d* = 0.77, 95%CI: 0.16, 1.38; n = 67) and HC users (adjusted mean = 0.71, 95% CI: 0.19 to 1.22; *P* = 0.021; *d* = 0.64, 95% CI: 0.02, 1.27; *n* = 70). Neither loading impact characteristics of the different endurance sports (*P* = *0*.158) nor athletic caliber (*P* = 0.066) were significant predictors in the total hip model. There were no significant predictors for either the lumbar spine or whole-body models. In isolation, lumbar spine BMD was higher in Tier 4–5 (0.5 ± 1.2, *n* = 77) vs Tier 2–3 (0.05 ± 1.1, *n* = 142, *P* = 0.002), while there were no group differences at total hip (*P* = 0.16) or whole-body (*P* = 0.11).

## Discussion

Among female athletes not using HC, the prevalence of severe MD was 37% in endurance, 34% in ball sport and 36% in power- and technical- athletes. The prevalence of low BMD at the lumbar spine was significantly higher among endurance athletes (17%) compared to non-endurance athletes (2%). No significant differences in BMD were observed at any site between women with severe MD, RM and HC users. Endurance athletes had significantly lower lumbar spine BMD Z-scores compared to non-endurance athletes. No differences were observed at total hip and whole-body level. Within endurance athletes, athletic caliber and loading impact characteristics of the different sports did not influence BMD at any site, but endurance athletes with severe MD showed lower total hip Z-scores compared to RM and HC users.

### Menstrual status

In this study, severe MD was reported in 34–37% of female athletes across the three sport categories. A review in female athletes of reproductive age reported the prevalence of severe MD to be in the range of 0–61%, with highest prevalence in leanness sports (Gimunova et al., 13. In contrast, our findings showed no such difference between endurance and non-endurance sports. One possible explanation is the way sports were categorized, as both the endurance and the power- and technical- categories in our study included leanness sports. Also, it should be acknowledged that three of the projects included in the current study recruited exclusively RM women, reporting a history of regular menstrual cycles. While sensitivity analysis excluding these three studies showed similar prevalence of severe MD across sport categories, the prevalence of severe MD could be underestimated and must be interpreted with caution. Furthermore, Taim et al. [41] pointed out heterogeneity across studies (assessment method, activity level, sport type, type and definition of MD) as the main reason for the wide range of prevalence estimates reported in the literature. The seven projects included in the current study used different assessment methods to define menstrual status, which could have impacted the results. Our findings underscore the importance of recognizing that severe MD can occur across a wide range of sports and not only in leanness sports. Furthermore, HC use could potentially mask MD, and as such the true prevalence of severe MD could be even higher.

In total, 49% of the women in the current study were currently using HC, which is somewhere in between the 29% reported among female athletes in Germany [21] and the 63–68% reported in Norway and Sweden [6, 8]. In the present study, the highest prevalence of HC use was observed in power- and technical- athletes (63%) and among the highest athletic caliber (Tier 4–5,58%). This is similar to the 55% HC use among elite athletes reported by Ekenros et al. [6]. However, they found the highest prevalence to be among sub-elite athletes (67%), which contrasts with our findings. Given the high prevalence of HC use among female athletes, differing between sports and countries, further research is warranted to investigate its effect on health and performance parameters. This is noteworthy, as 15–16% of HC users report using HC to manage irregular menstrual cycles [6, 21], which contrasts with the Endocrine Society’s recommendations [14]. As LEA can cause irregular cycles, the use of HC to manage these could mask LEA, and thus LEA cannot be confirmed or disproved in athletes using HC [19].

### Bone health

Among the 221 athletes with BMD data, the highest prevalence of low BMD was observed in endurance athletes at the lumbar spine (17%), whereas the lowest prevalence was found in non-endurance athletes at the lumbar spine (2%). Tenforde et al. [42] reported that 10% of athletes had low BMD at the whole body and/or lumbar spine, with the highest prevalence observed in athletes participating in synchronized swimming (45%), followed by swimming (24%) and cross-country running (19%), and no low BMD in athletes from high-impact sports such as basketball, softball, gymnastics, and lacrosse. In the current study, endurance athletes showed lower lumbar spine BMD Z-scores compared to non-endurance athletes. Similarly, Hinrichs et al. [17] also reported lower lumbar spine BMD in endurance athletes compared to team sport and power athletes.

A significant group × age interaction was observed for total hip BMD, indicating that BMD declined significantly with age in non-endurance athletes, while no significant age-related trend was observed among endurance athletes. Notably, non-endurance athletes exhibited higher hip BMD at younger ages; however, the between-group difference was no longer significant from 27 years onward. These findings suggest that differences in total hip BMD between endurance and non-endurance athletes are more pronounced at younger ages and diminish with increasing age. This may partly explain inconsistencies across previous studies with differing age distributions and supports the notion that skeletal adaptations and/or bone maintenance may differ between sport groups across the lifespan.

Less clear differences between sports for BMD at the hip than lumbar spine may be explained by the nature of mechanical loading on the body. Thirty-three percent of our endurance athletes were runners, where the ground reaction forces are gradually reduced from the foot upwards, and the load on the lumbar spine may not be high enough to stimulate bone adaptation. In contrast, the higher forces generated in power sports are more likely to reach this threshold, which could explain why we observed differences for lumbar spine BMD without differences for the hip. For the non-endurance athletes that represented ball sport and power- and technical- sports, it can be argued that dynamic, weight-bearing activities with high force magnitude and speed (ball and power sports) provide a stronger stimulus for bone adaptation [46]. However, as impact classification only was performed for the endurance sports (due to smaller number of participants per sport and large variation in proposed impact of the non-endurance sports), we can only speculate on whether mechanical loading could be a possible explanation for differences between sport categories. The observed differences in BMD between sport categories further support the conclusion by Jonvik et al. [20] that defining low BMD in athletes requires sport-specific reference values.

Only one individual had low whole-body BMD, which is less than expected, as a normal distribution curve would predict that approximately 16% of a population would have low BMD. Accordingly, no difference in whole-body BMD was found between endurance athletes and non-endurance athletes. The trabecular bones in lumbar spine and total hip are more sensitive sites, in which earlier signs of catabolic conditions can be apparent, while low whole-body BMD can be an indication that the condition has become very severe (Lesley & Binkley, 22. In line, those who reported a history of bone stress injury had lower lumbar spine BMD Z-scores than those who reported no history of bone stress injuries, with no differences at whole-body level. Similarly, a smaller study on female runners found that those with a history of high-risk bone stress injury had lower BMD at lumbar spine only [43]. High-risk bone stress injury is listed as a red flag in the 2023 IOC REDs consensus [27], and identification of site-specific low BMD as a risk factor could be important for bone stress injury prevention.

The BMD findings should be interpreted considering the grouping strategy used, as pooling athletes from multiple disciplines may have attenuated sport-specific loading effects compared with studies focusing on individual sports. Sport-specific sub-analyses were not performed due to limited sample sizes and risk of underpowered analyses.

### Menstrual status and bone health

There was no difference in BMD Z-scores between severe MD, RM and HC users at any site. It was expected that athletes with severe MD would show lower BMD, given the beneficial effects of estrogen and progesterone on bone [38] and previous reports of low BMD in female athletes with severe MD (Tenforde, 43. One possible explanation is that the RM group likely included some athletes with subtle MD, such as anovulation or luteal phase deficiency, conditions associated with reduced ovarian hormone levels [36], which potentially could have masked between-group differences. Among athletes with severe MD, the duration of the condition was not documented, and it may have been insufficient to exert a measurable impact on BMD. At the individual level, a 1–2-year interval between BMD assessments is generally recommended to detect clinically meaningful changes [39]. Retrospective studies, however, have shown that athletes with a history of amenorrhea or oligomenorrhea may experience lower BMD later in life [30], and as such athletes with current severe MD might not yet have developed low BMD.

The lack of significant differences in BMD between severe MD, RM and HC users may be attributed to the inclusion of both combined hormonal contraceptives and progestin-only within the HC group. Moreover, the duration of HC use may also have influenced the results. However, information about the duration of HC use was not available in the current dataset. Injectable progestin-only contraceptives have been shown to negatively affect BMD, whereas hormonal IUD and hormonal implants appear to exert a neutral or potentially positive effect [9]. Previous research has reported lower BMD Z-scores among oral contraceptive pill users compared to non-HC users, suggesting an adverse effect on bone health [2, 4]. The use of the oral contraceptive pill during adolescence may further impair BMD due to suppression of endogenous hormone production [5]. It should also be considered that within the cohort of HC users, there might be a subset of women that suffers from LEA, which represents an additional factor potentially contributing to compromised BMD. Although no differences were observed in the current study, the collective evidence indicates that the impact of HC on BMD likely varies according to contraceptive type, hormonal dose, route of administration, and the user’s age and endogenous hormonal milieu [5, 9].

### Factors influencing bone mineral density in endurance athletes

The final study aim was to explore factors that could influence BMD within endurance athletes, using predictor models. Neither sport-specific impact classification nor athletic caliber influenced BMD at any site. We did, however, observe lower total hip BMD in endurance athletes with severe MD compared to those with RM or HC use. The association between severe MD and low BMD has also been observed in several other previous studies Gimunova et al., 2024), [1, 29]. A common consequence of LEA is MD, and endurance athletes are particularly vulnerable due to their high energy expenditure [26] and the competitive advantage of low body mass for improved work economy. LEA may reduce BMD by limiting the energy available for bone maintenance and adaptation, suggesting that LEA, alone or in combination with MD, could contribute to lower BMD [27]. However, whether the lower hip BMD observed in endurance athletes with severe MD is directly related to LEA cannot be determined in this retrospective analysis of cross-sectional data, as we do not have data on energy availability or a full REDs assessment. Nevertheless, our findings highlight the importance of preventing severe MD in endurance athletes as a key strategy for optimizing bone health.

In contrast to the findings at the total hip, no differences in lumbar spine BMD were observed across menstrual status categories. It could be speculated that total hip BMD comprises a higher proportion of cortical bone, which appears to be more sensitive to chronic estrogen exposure, whereas lumbar spine BMD is predominantly trabecular. Thus, menstrual disturbances and associated alterations in estrogen levels may preferentially affect hip BMD [10]. It may also reflect the generally lower lumbar spine BMD across the cohort, particularly among endurance athletes, where lower osteogenic loading may overshadow potential effects of menstrual disturbances [18].

Even though we were unable to detect any effect of sport-specific impact classification, this could be a question of lack of information on actual impact loading per sport and per participant. Unfortunately, we do not have insight into the participants’ training programs or strength training background. A review from 2012 noted that, due to differences in loading forces and energy expenditure, distance runners, cyclists and swimmers generally exhibit poorer bone health than athletes from other sports [37]. This previous literature has most often compared BMD between one or two specific sports with clearly distinct loading characteristics. The current study presents a broad selection of endurance athletes from 13 different sports, whereof 10 categorized as medium, low and very-low impact. The heterogeneity of sports loading within each category could influence the results.

When examined in isolation, lumbar spine BMD was higher in Tier 4–5 compared with Tier 2–3 endurance athletes, however this finding was not supported when athletic caliber was included in multivariable predictor models for BMD at any site. Similarly, Hilkens et al. [16] reported comparable lumbar spine and total hip BMD in female cyclists at early and advanced career stages. To our knowledge, no previous study has examined the association between BMD and athletic caliber across different sports. It therefore remains unclear whether a potential influence of athletic caliber on BMD is masked by stronger determinants of bone health, such as sport-specific mechanical loading and exposure to strength training, which were not fully captured in the current study.

### Methodological considerations

The main strength of this study is the big dataset of 411 women from 34 different sports, with comprehensive data on menstrual status, athletic caliber and BMD (*n* = 211). This broad sample allowed us to examine the prevalence of severe MD and low BMD, as well as factors related to bone health in a diverse group of female athletes. Some limitations of this study should also be acknowledged. Firstly, the grouping of multiple sports into broad categories represents a methodological limitation, as it may mask sport-specific effects on BMD. However, this approach was necessary given the study aims and available sample size. Secondly, three of the included studies exclusively recruited athletes with a menstrual cycle length between 21 and 35 days, thereby excluding women with severe MD. However, a sensitivity analysis excluding these studies showed no relevant changes in prevalence estimates of severe MD. Nonetheless, the prevalence of severe MD should still be interpreted cautiously, as heterogeneity in study design and menstrual assessment methods may influence classification. As the current study was initiated after data completion of the included studies, it was not possible to incorporate the same methodological procedures for assessing menstrual status across studies. This may increase the risk of misclassification of participants into incorrect menstrual status categories. To reduce this risk, participants were grouped into three main categories, RM, severe MD and HC users. However, the presence of athletes with unrecognized subtle MD within the RM group may obscure the true impact of menstrual status on BMD. Thirdly, the use of different DXA machines across studies may have influenced the results. As this dataset was retrospectively compiled from multiple independent laboratories, formal harmonization between DXA systems (e.g., Lunar and Hologic) was not feasible given limited access to raw calibration data across sites. We also note that, although several cross-calibration equations between DXA manufacturers have been proposed in the literature [11, 32, 33], there is an ongoing debate on their reliability across different measurement sites, populations, and scanning protocols. For this reason, such conversions were not applied. However, we have accommodated this by including DXA machine as a random intercept in our statistical models. Lastly, the grouping of endurance sports by impact loading was solely based on the type of sport, and we did not have insight into the type of training performed by the individual athletes. As the majority of participants were endurance athletes, and previous literature provides clearer frameworks for impact classification within endurance sports (e.g., [46], impact was classified for endurance sports only. Consequently, analyses investigating factors influencing BMD (aim 3) were restricted to endurance athletes only, which should be considered when interpreting these findings.

### Practical implications

The present findings emphasize the need for sports organizations, coaches, and medical support teams to recognize athletes as a particularly vulnerable group for severe MD, with endurance athletes showing additional risk of reduced BMD. Preventive strategies should include systematic monitoring of menstrual function and bone health, alongside education on adequate energy availability and its importance in long-term performance and health. In practice, this requires the implementation of structured screening protocols and multidisciplinary management approaches (e.g., REDs clinical assessment tool version 2) to enable early detection and targeted intervention [27], thereby reducing health risks while supporting sustainable athletic performance [15].

Prospective longitudinal studies that incorporate comprehensive REDs assessments alongside detailed data on BMD, menstrual status, training history, bone-loading impact and injury history are needed to elucidate causal pathways and to inform targeted prevention and management strategies.

## Conclusion

This study demonstrated a high prevalence of severe MD among female athletes, with no differences across endurance, ball sport, and power- and technical- sports. BMD Z-scores did not differ between athletes with severe MD, RM, and HC users. In contrast, endurance athletes demonstrated lower BMD Z-scores than non-endurance athletes, most notably among those with severe MD. Heterogenous menstrual assessment methods across the projects included in this study could impact the results. The absence of an association between impact loading in endurance sports and BMD suggests that mechanical load alone is insufficient to explain the observed differences.

## Supplementary Information

Supplementary Material 1.

## Data Availability

Availability of the data from the individual projects is described in the respective trial registrations and/or publications listed in Table 1. The total dataset used during the current study is available from the corresponding author on reasonable request.

## Abbreviations

BMD: Bone mineral density
DXA: Dual-energy X-ray Absorptiometry
HC: Hormonal contraceptives
LEA: Low energy availability
MD: Menstrual disturbances
REDs: Relative energy deficiency in sport
RM: Regularly menstruating

## References

[1] Ackerman KE, Nazem T, Chapko D, Russell M, Mendes N, Taylor AP, et al. Bone microarchitecture is impaired in adolescent amenorrheic athletes compared with eumenorrheic athletes and nonathletic controls. J Clin Endocrinol Metab. 2011;96(10):3123–33. 10.1210/jc.2011-1614. 21816790 10.1210/jc.2011-1614 PMC3200253

[2] Ackerman KE, Singhal V, Baskaran C, Slattery M, Campoverde Reyes KJ, Toth A, et al. Oestrogen replacement improves bone mineral density in oligo-amenorrhoeic athletes: a randomised clinical trial. Br J Sports Med. 2019;53(4):229–36. 10.1136/bjsports-2018-099723. 30301734 10.1136/bjsports-2018-099723 PMC6686188

[3] Allaway HC, Southmayd EA, De Souza MJ. The physiology of functional hypothalamic amenorrhea associated with energy deficiency in exercising women and in women with anorexia nervosa. Horm Mol Biol Clin Investig. 2016;25(2):91–119. 10.1515/hmbci-2015-0053. 26953710 10.1515/hmbci-2015-0053

[4] Almstedt HC, Cook MM, Bramble LF, Dabir DV, LaBrie JW. Oral contraceptive use, bone mineral density, and bone turnover markers over 12 months in college-aged females. J Bone Miner Metab. 2020;38(4):544–54. 10.1007/s00774-019-01081-1. 31983034 10.1007/s00774-019-01081-1 PMC10317246

[5] Ampatzis C, Zervoudis S, Iatrakis G, Mastorakos G. Effect of oral contraceptives on bone mineral density. Acta Endocrinol (Buchar). 2022;18(3):355–60. 10.4183/aeb.2022.355. 36699167 10.4183/aeb.2022.355 PMC9867809

[6] Ekenros L, von Rosen P, Solli G, Sandbakk Ø, Holmberg H-C, Hirschberg A, et al. Perceived impact of the menstrual cycle and hormonal contraceptives on physical exercise and performance in 1,086 athletes from 57 sports. Front Physiol. 2022. 10.3389/fphys.2022.954760. 36111164 10.3389/fphys.2022.954760 PMC9468598

[7] Elliott-Sale KJ, Minahan CL, de Jonge X, Ackerman KE, Sipilä S, Constantini NW, et al. Methodological considerations for studies in sport and exercise science with women as participants: a working guide for standards of practice for research on women. Sports Med. 2021;51(5):843–61. 10.1007/s40279-021-01435-8. 33725341 10.1007/s40279-021-01435-8 PMC8053180

[8] Engseth TP, Andersson EP, Solli GS, Morseth B, Thomassen TO, Noordhof DA, et al. Prevalence and self-perceived experiences with the use of hormonal contraceptives among competitive female cross-country skiers and biathletes in Norway: The FENDURA Project. Front Sports Act Living. 2022;4:873222. 10.3389/fspor.2022.873222. 35498528 10.3389/fspor.2022.873222 PMC9047044

[9] Erhardt-Ohren B, Prata N, Rosenblum S. Bone mineral density changes during use of progestin-only contraceptives: a rapid review of recent evidence. AJOG Glob Rep. 2025;5(3):100509. 10.1016/j.xagr.2025.100509. 40607241 10.1016/j.xagr.2025.100509 PMC12213273

[10] Farr JN, Khosla S, Miyabara Y, Miller VM, Kearns AE. Effects of estrogen with micronized progesterone on cortical and trabecular bone mass and microstructure in recently postmenopausal women. J Clin Endocrinol Metab. 2013;98(2):E249–57. 10.1210/jc.2012-3406. 23322818 10.1210/jc.2012-3406 PMC3565106

[11] Faulkner KG, Roberts LA, McClung MR. Discrepancies in normative data between Lunar and Hologic DXA systems. Osteoporos Int. 1996;6(6):432–6. 9116387 10.1007/BF01629574

[12] Furu K, Aares EB, Hjellvik V, Karlstad Ø. Hormonal contraceptive use in Norway, 2006–2020, by contraceptive type, age and county: a nationwide register-based study. Norsk Epidemiol. 2021;29(1–2):1–2. 10.5324/nje.v29i1-2.4046.

[13] Gimunová M, Paulínyová A, Bernaciková M, Paludo AC. The prevalence of menstrual cycle disorders in female athletes from different sports disciplines: a rapid review. Int J Environ Res Public Health. 2022. 10.3390/ijerph192114243. 36361122 10.3390/ijerph192114243 PMC9658102

[14] Gordon CM, Ackerman KE, Berga SL, Kaplan JR, Mastorakos G, Misra M, et al. Functional hypothalamic amenorrhea: an Endocrine Society clinical practice guideline. J Clin Endocrinol Metab. 2017;102(5):1413–39. 10.1210/jc.2017-00131. 28368518 10.1210/jc.2017-00131

[15] Heikura IA, McCluskey WTP, Tsai MC, Johnson L, Murray H, Mountjoy M, et al. Application of the IOC Relative Energy Deficiency in Sport (REDs) clinical assessment tool version 2 (CAT2) across 200+ elite athletes. Br J Sports Med. 2024. 10.1136/bjsports-2024-108121. 39164063 10.1136/bjsports-2024-108121

[16] Hilkens L, Van Schijndel N, Weijer V, Boerboom M, Van Der Burg E, Peters V, et al. Low bone mineral density and associated risk factors in elite cyclists at different stages of a professional cycling career. Med Sci Sports Exerc. 2023;55(5):957–65. 10.1249/MSS.0000000000003113. 36595659 10.1249/MSS.0000000000003113 PMC10090358

[17] Hinrichs T, Chae E-H, Lehmann R, Allolio B, Platen P. Bone mineral density in athletes of different disciplines: a cross- sectional study. Open Sports Sci J. 2010;3:129–33.

[18] Hutson MJ, O’Donnell E, Brooke-Wavell K, Sale C, Blagrove RC. Effects of low energy availability on bone health in endurance athletes and high-impact exercise as a potential countermeasure: a narrative review. Sports Med. 2021;51(3):391–403. 10.1007/s40279-020-01396-4. 33346900 10.1007/s40279-020-01396-4 PMC7900047

[19] Ihalainen J, Mikkonen R, Ackerman K, Heikura I, Mjøsund K, Valtonen M, et al. Beyond menstrual dysfunction: does altered endocrine function caused by problematic low energy availability impair health and sports performance in female athletes? Sports Med. 2024;54:2267–89. 10.1007/s40279-024-02065-6. 38995599 10.1007/s40279-024-02065-6 PMC11393114

[20] Jonvik KL, Torstveit MK, Sundgot-Borgen J, Mathisen TF. Do we need to change the guideline values for determining low bone mineral density in athletes? J Appl Physiol. 2022;132(5):1320–2. 10.1152/japplphysiol.00851.2021. 35060767 10.1152/japplphysiol.00851.2021 PMC9126212

[21] Kirschbaum EM, Fischer K, Speiser D, Lautenbach F, Schwenkreis F, Dathan-Stumpf A, et al. Prevalence of menstrual dysfunction and hormonal contraceptive use among elite female athletes from different sports in Germany. Sports Medicine – Open. 2025;11(1):49. 10.1186/s40798-025-00845-6. 40332721 10.1186/s40798-025-00845-6 PMC12058627

[22] Leslie WD, Binkley N. Spine Bone Texture and the Trabecular Bone Score (TBS). In: Patel V, Preedy V. (eds) Biomarkers in Bone Disease. Biomarkers in Disease: Methods, Discoveries and Applications. Springer, Dordrecht. 2017. 10.1007/978-94-007-7693-7_33.

[23] Lindh I, Skjeldestad FE, Gemzell-Danielsson K, Heikinheimo O, Hognert H, Milsom I, et al. Contraceptive use in the Nordic countries. Acta Obstet Gynecol Scand. 2017;96(1):19–28. 10.1111/aogs.13055. 27861709 10.1111/aogs.13055

[24] McKay AKA, Stellingwerff T, Smith ES, Martin DT, Mujika I, Goosey-Tolfrey VL, et al. Defining training and performance caliber: a participant classification framework. Int J Sports Physiol Perform. 2022;17(2):317–31. 10.1123/ijspp.2021-0451. 34965513 10.1123/ijspp.2021-0451

[25] Melin A, Tornberg ÅB, Skouby S, Faber J, Ritz C, Sjödin A, et al. The LEAF questionnaire: a screening tool for the identification of female athletes at risk for the female athlete triad. Br J Sports Med. 2014;48:540–5. 10.1136/bjsports-2013-093240. 24563388 10.1136/bjsports-2013-093240

[26] Melin A, Tornberg ÅB, Skouby S, Møller SS, Sundgot-Borgen J, Faber J, et al. Energy availability and the female athlete triad in elite endurance athletes. Scand J Med Sci Sports. 2015;25(5):610–22. 10.1111/sms.12261. 24888644 10.1111/sms.12261

[27] Mountjoy M, Ackerman KE, Bailey DM, et al. 2023 International Olympic Committee’s (IOC) consensus statement on Relative Energy Deficiency in Sport (REDs). Br J Sports Med. 2023;57(17):1073–97. 10.1136/bjsports-2023-106994. 37752011 10.1136/bjsports-2023-106994

[28] Nattiv A, Loucks AB, Manore MM, Sanborn CF, Sundgot-Borgen J, Warren MP. American College of Sports Medicine. American College of Sports Medicine position stand. The female athlete triad. Med Sci Sports Exerc. 2007;39(10):1867–82. 10.1249/mss.0b013e318149f111. 17909417 10.1249/mss.0b013e318149f111

[29] Nichols JF, Rauh MJ, Barrack MT, Barkai HS. Bone mineral density in female high school athletes: Interactions of menstrual function and type of mechanical loading. Bone. 2007;41(3):371–7. 10.1016/j.bone.2007.05.003. 17572167 10.1016/j.bone.2007.05.003

[30] Nose-Ogura S, Yoshino O, Dohi M, Kigawa M, Harada M, Kawahara T, et al. Low bone mineral density in elite female athletes with a history of secondary amenorrhea in their teens. Clin J Sport Med. 2020;30(3):245–50. 10.1097/jsm.0000000000000571. 32341292 10.1097/JSM.0000000000000571

[31] Osborne J, Storvand J, Engseth T, Solli G, Morseth B, Taylor M, Welde B, Elliott‐Sale K, Andersson E, Sandbakk O & Noordhof D. Prevalence of hormonal contraceptive use and self‐reported symptomatic experiences attributed to the menstrual cycle or hormonal contraceptive use in Norwegian women: The effect of training categories and age groups ‐ The FENDURA Project. Scand J Med Sci Sports. 2025;35. 10.1111/sms.70096. 10.1111/sms.70096 PMC12254879 40650410

[32] Ozdemir A, Uçar M. Standardization of spine and hip BMD measurements in different DXA devices. Eur J Radiol. 2007;62(3):423–6. 17289322 10.1016/j.ejrad.2006.11.034

[33] Pearson D, Cawte SA, Green DJ. A comparison of phantoms for cross-calibration of lumbar spine DXA. Osteoporos Int. 2002;13(12):948–54. 12459937 10.1007/s001980200132

[34] R Core Team. R: A language and environment for statistical computing. R Foundation for Statistical Computing; 2024.

[35] Schaumberg MA, Jenkins DG, Janse de Jonge XAK, Emmerton LM, Skinner TL. Three-step method for menstrual and oral contraceptive cycle verification. J Sci Med Sport. 2017;20(11):965–9. 10.1016/j.jsams.2016.08.013. 28684053 10.1016/j.jsams.2016.08.013

[36] Schliep KC, Mumford SL, Hammoud AO, et al. Luteal phase deficiency in regularly menstruating women: prevalence and overlap in identification based on clinical and biochemical diagnostic criteria. J Clin Endocrinol Metab. 2014;99(6):E1007–14. 10.1210/jc.2013-3534. 24606080 10.1210/jc.2013-3534 PMC4037737

[37] Scofield KL, Hecht S. Bone health in endurance athletes: runners, cyclists, and swimmers. Curr Sports Med Rep. 2012;11(6):328–34. 10.1249/JSR.0b013e3182779193. 23147022 10.1249/JSR.0b013e3182779193

[38] Seifert-Klauss V, Prior JC. Progesterone and bone: actions promoting bone health in women. J Osteoporos. 2010;2010(1):845180. 10.4061/2010/845180. 21052538 10.4061/2010/845180 PMC2968416

[39] Shuhart C, Cheung A, Gill R, Gani L, Goel H, Szalat A. Executive summary of the 2023 Adult Position Development Conference of the International Society for Clinical Densitometry: DXA reporting, follow-up BMD testing and trabecular bone score application and reporting. J Clin Densitom. 2024;27(1):101435. 10.1016/j.jocd.2023.101435. 38007332 10.1016/j.jocd.2023.101435

[40] Southmayd EA, Williams NI, Mallinson RJ, De Souza MJ. Energy deficiency suppresses bone turnover in exercising women with menstrual disturbances. J Clin Endocrinol Metab. 2019;104(8):3131–45. 10.1210/jc.2019-00089. 30896746 10.1210/jc.2019-00089

[41] Taim BC, Ó Catháin C, Renard M, Elliott-Sale KJ, Madigan S & Ní Chéilleachair N. The prevalence of menstrual cycle disorders and menstrual cycle-related symptoms in female athletes: A systematic literature review. Sports Med. 2023;53(10):1963–1984. https://link.springer.com/article/10.1007/s40279-023-01871-8. 10.1007/s40279-023-01871-8 37389782

[42] Tenforde AS, Carlson JL, Sainani KL, Chang AO, Kim JH, Golden NH, et al. Sport and triad risk factors influence bone mineral density in collegiate athletes. Med Sci Sports Exerc. 2018;50(12):2536–43. 10.1249/mss.0000000000001711. 29975299 10.1249/MSS.0000000000001711

[43] Tenforde AS, Ackerman KE, Bouxsein ML, et al. Factors associated with high-risk and low-risk bone stress injury in female runners: implications for risk factor stratification and management. Orthop J Sports Med. 2024;12(5):23259671241246227. 10.1177/23259671241246227. 38779133 10.1177/23259671241246227 PMC11110515

[44] Torstveit MK, Sundgot-Borgen J. Participation in leanness sports but not training volume is associated with menstrual dysfunction: a national survey of 1276 elite athletes and controls. Br J Sports Med. 2005;39(3):141–7. 10.1136/bjsm.2003.011338. 15728691 10.1136/bjsm.2003.011338 PMC1725151

[45] Tønnessen E, Sandbakk Ø, Sandbakk SB, Seiler S, Haugen T. Training session models in endurance sports: a Norwegian perspective on best practice recommendations. Sports Med. 2024;54(11):2935–53. 10.1007/s40279-024-02067-4. 39012575 10.1007/s40279-024-02067-4 PMC11560996

[46] Weeks BK, Beck BR. The BPAQ: a bone-specific physical activity assessment instrument. Osteoporos Int. 2008;19(11):1567–77. 10.1007/s00198-008-0606-2. 18414964 10.1007/s00198-008-0606-2

