# Supplementary material for: Menstrual status and bone health in athletes from different sports – a retrospective analysis
Source: BMC Sports Sci Med Rehabil. 2026 Jun 9;18:356. doi: 10.1186/s13102-026-01781-y (PMC13474736; doi:10.1186/s13102-026-01781-y)
Supplement: Supplementary file 1 [file 13102_2026_1781_MOESM1_ESM.docx]

**Supplemental Figure 1.** Bone mineral density of endurance athletes, grouped by menstrual status. Individual colored points represent raw, unadjusted participant data for (A) whole-body (n=74), (B) total hip (n=179), and (C) lumbar spine (n=180). Black points indicate model-adjusted means for each group with black vertical lines showing 95% confidence intervals, derived from a linear mixed-effects model adjusted for age, BMI, loading impact characteristics, athletic caliber and DXA machine (random intercept). Z-scores are unitless standardized values representing the number of standard deviations above or below the population mean for age and sex. *Indicates a statistically significant lower *z*-score than other groups (*P*<0.05).
